# Author Correction: Fundamental investigations on the sodium-ion transport properties of mixed polyanion solid-state battery electrolytes

**DOI:** 10.1038/s41467-022-33224-w

**Published:** 2022-09-21

**Authors:** Zeyu Deng, Tara P. Mishra, Eunike Mahayoni, Qianli Ma, Aaron Jue Kang Tieu, Olivier Guillon, Jean-Noël Chotard, Vincent Seznec, Anthony K. Cheetham, Christian Masquelier, Gopalakrishnan Sai Gautam, Pieremanuele Canepa

**Affiliations:** 1 Department of Materials Science and Engineering, National University of Singapore, Singapore, 117575 Singapore; 2 Singapore-MIT Alliance for Research and Technology, 1 CREATE Way, 10-01 CREATE Tower, Singapore, 138602 Singapore; 3 Laboratoire de Réactivité et de Chimie des Solides (LRCS), CNRS UMR 7314, Université de Picardie Jules Verne, 80039 Amiens, Cedex 1 France; 4 RS2E, Réseau Français sur le Stockage Electrochimique de l’Energie, FR CNRS 3459, F-80039 Amiens, Cedex 1 France; 5 ALISTORE-ERI European Research Institute, FR CNRS 3104, Amiens, F-80039 Cedex 1 France; 6 Forschungszentrum Jülich GmbH, Institute of Energy and Climate Research, Materials Synthesis and Processing (IEK-1), 52425 Jülich, Germany; 7 Helmholtz-Institute Münster, c/o Forschungszentrum Jülich GmbH, 52425 Jülich, Germany; 8 Materials Department and Materials Research Laboratory, University of California, Santa Barbara, 93106 CA USA; 9 Department of Materials Engineering, Indian Institute of Science, Bengaluru, 560012 Karnataka India; 10 Chemical and Biomolecular Engineering, National University of Singapore, 4 Engineering Drive 4, Singapore, 117585 Singapore

**Keywords:** Batteries, Energy storage, Atomistic models, Solid-state chemistry

Correction to: *Nature Communications* 10.1038/s41467-022-32190-7, published online 02 August 2022

In the original version of this article, one of the affiliation details for Anthony K. Cheetham were incorrectly given as ‘Materials Department and Materials Research Laboratory, University of California, Santa Barbara, Bengaluru 93106 California, USA’ but should have been ‘Materials Department and Materials Research Laboratory, University of California, Santa Barbara, 93106 California, USA’. Also, in the original version of the Supplementary Information associated with this Article, the affiliations of Anthony K. Cheetham, Gopalakrishnan Sai Gautam and Pieremanuele Canepa were incorrectly reported and numbered. Also, this article contained mistyping errors in Figure 2, Figure 4, Supplementary Table 1, Supplementary Table 4 and Supplementary Note 3. In Figure 2, panel b, the y-axis label was incorrectly reported as “Na+ conductivity, log10() (S/cm)”. In Figure 4, panel a, the x-axis label was incorrectly reported as “2 (°)”. In Figure 4, panel b, the y-axis label was incorrectly reported as “ln(T) (SKcm-1)”. In Supplementary Table 1, the numerical values “623.9” and “625.2” in the “AE_end_” column were missing the “-” sign, and the terms “Na(1)” and “Na(2)” were swapped. In Supplementary Table 4, the number “8” was used for two different “Cluster #” rows. In Supplementary Note 3, the terms “Na(1)” and “Na(2)” were swapped, and the term ΔE_end_ was incorrectly reported as “|ΔE_end_|”. The correct affiliations, axis labels, numerical values of tables and labelling of equation components are now provided in the main text and supplementary information. These errors have been corrected in the HTML and PDF versions of the article.

## Supplementary information

Updated Supplementary Information

