# Supplementary material for: Fundamental investigations on the sodium-ion transport properties of mixed polyanion solid-state battery electrolytes
Source: Nat Commun. 2022 Aug 2;13:4470. doi: 10.1038/s41467-022-32190-7 (PMC9345873; doi:10.1038/s41467-022-32190-7)
Supplement: Supplementary file 1 — Supplementary information [file 41467_2022_32190_MOESM1_ESM.pdf]

Supplementary Information

Fundamental Investigations on the Sodium-Ion Transport Properties of Mixed Polyanion Solid-State Battery Electrolytes

Zeyu Deng,[1*] Tara P. Mishra,[1,2] Eunike Mahayoni,[3,4,5] Qianli Ma,[6] Aaron Jue Kang Tieu,[1] Olivier Guillon,[6,7] Jean-Noël Chotard,[3,4,5] Vincent Seznec,[3,4,5] Anthony K. Cheetham,[1,8] Christian Masquelier,[3,4,5] Gopalakrishnan Sai Gautam,[9] and Pieremanuele Canepa[1,2,10 #]

[1]Department of Materials Science and Engineering,
National University of Singapore, Singapore 117575, Singapore
[2]Singapore-MIT Alliance for Research and Technology, 1 CREATE Way,
10-01 CREATE Tower, Singapore 138602, Singapore
[3]Laboratoire de Réactivité et de Chimie des Solides (LRCS), CNRS UMR 7314,
Université de Picardie Jules Verne, 80039 Amiens Cedex, France.
[4]RS2E, Réseau Français sur le Stockage Electrochimique de l'Energie,
FR CNRS 3459, F-80039 Amiens Cedex 1, France.
[5]ALISTORE-ERI European Research Institute, FR CNRS 3104, Amiens, F-80039 Cedex 1, France.
[6]Forschungszentrum Jülich GmbH, Institute of Energy and Climate Research,
Materials Synthesis and Processing (IEK-1), 52425 Jülich, Germany
[7]Helmholtz-Institute Münster, c/o Forschungszentrum Jülich GmbH, 52425 Jülich, Germany
[8]Materials Department and Materials Research Laboratory, University of California, Santa Barbara, 93106 California, USA.
[9]Department of Materials Engineering,
Indian Institute of Science, Bengaluru, 560012, Karnataka, India
[10]Chemical and Biomolecular Engineering,
National University of Singapore, 4 Engineering Drive 4, Singapore, 117585, Singapore.

Corresponding authors: *msedz@nus.edu.sg and #pcanepa@nus.edu.sg

Supplementary Figures

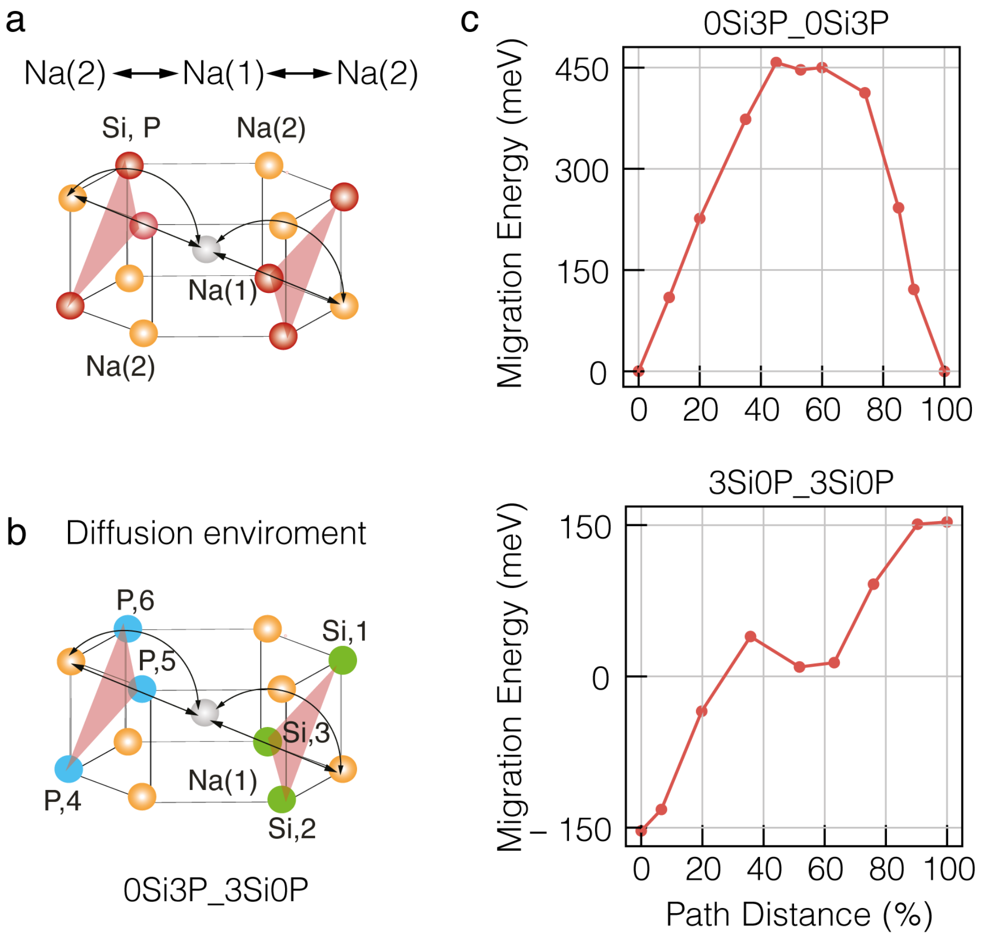

Supplementary Figure 1. Panel a Migration unit and migration events indicated as first Na(2)→Na(1) and second Na(1)→Na(2). The Na(1) site is indicated in grey, whereas (Na) sites are colored in orange. Panel b migration unit emphasizing the diffusion environment 0Si3P_3Si0P, with P and Si atoms indicated as light-blue and green, respectively. Panel c migration barriers of Na atom from (left) Na(1) site to adjacent Na(1) site via a Na(2) site for a x = 0 and (right) Na(2) to Na(1) site for a x = 3 in $Na_{1+x}Zr_2Si_xP_{3-x}O_{12}$ structure. The naming convention αSiβP_γSiδP is used. Migration energies are adjusted so that the average energies of the initial and final images are 0.

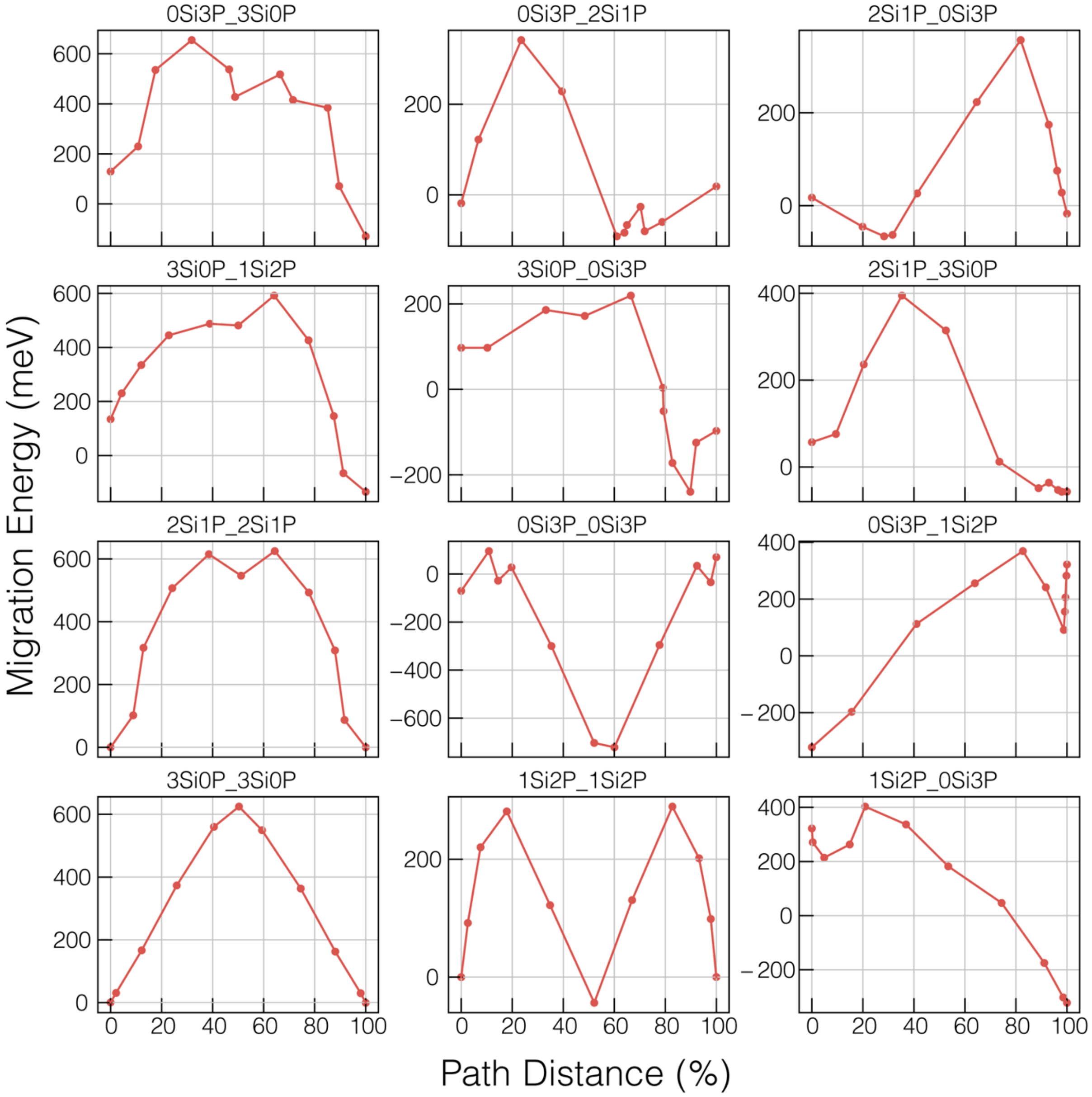

Supplementary Figure 2. NEB migration barriers form the Na(2) site to an adjacent Na(2) site via a Na(1) site for x = 2 in $Na_{1+x}Zr_2Si_xP_{3-x}O_{12}$ structure at different Si/P environment. The naming convention αSiβP_γSiδP is used. While charge neutrality is globally maintained for all barriers, local charge neutrality is not enforced. Migration energies are adjusted so that the average energies of the initial and final images are 0.

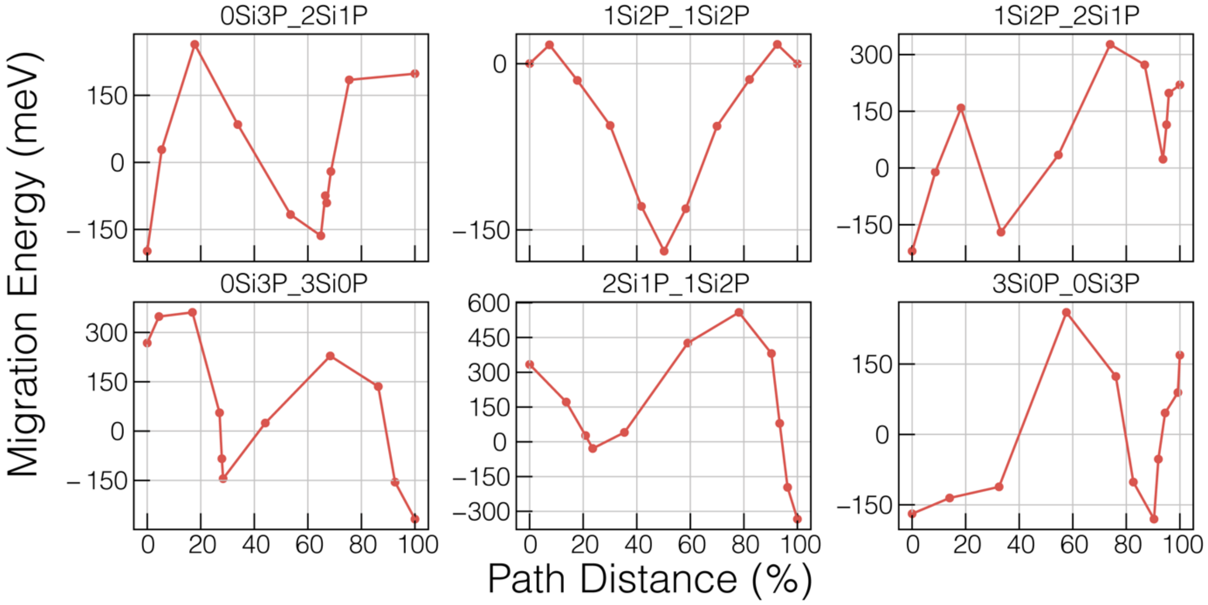

Supplementary Figure 3. NEB computed migration barrier form the Na(2) site to an adjacent Na(2) site via a Na(1) site for x = 2 in $Na_{1+x}Zr_2Si_xP_{3-x}O_{12}$ structure at different Si/P environment. These configurations maintain both global and local charge neutrality by removing extra Na atoms from the migration unit of Figure 1d in the main text. The naming convention αSiβP_γSiδP is used. Migration energies are adjusted so that the average energies of the initial and final images are 0.

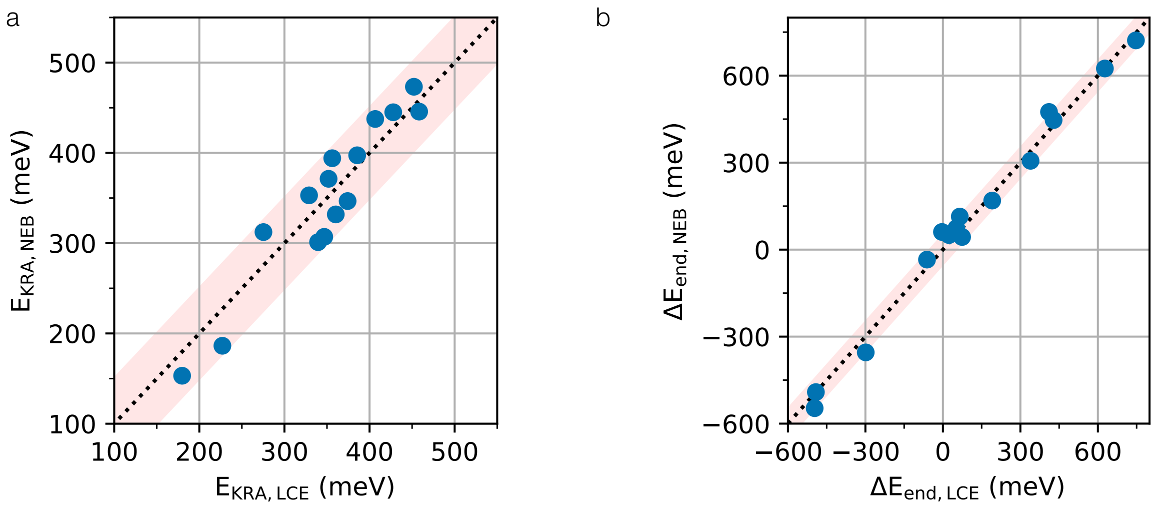

Supplementary Figure 4. Comparison between the predicted (y-axis) and calculated E_{KRA} (a) and ΔE_{end} (b) (x-axis) shown as blue dots. Pink ribbons indicate tolerable bounds (± 50 meV) in reproducing computed values of E_{KRA} and ΔE_{end}. The root mean square error was found to be ± 29.15 meV and ± 38.0 meV for E_{KRA} and ΔE_{end} local cluster expansion models, respectively.

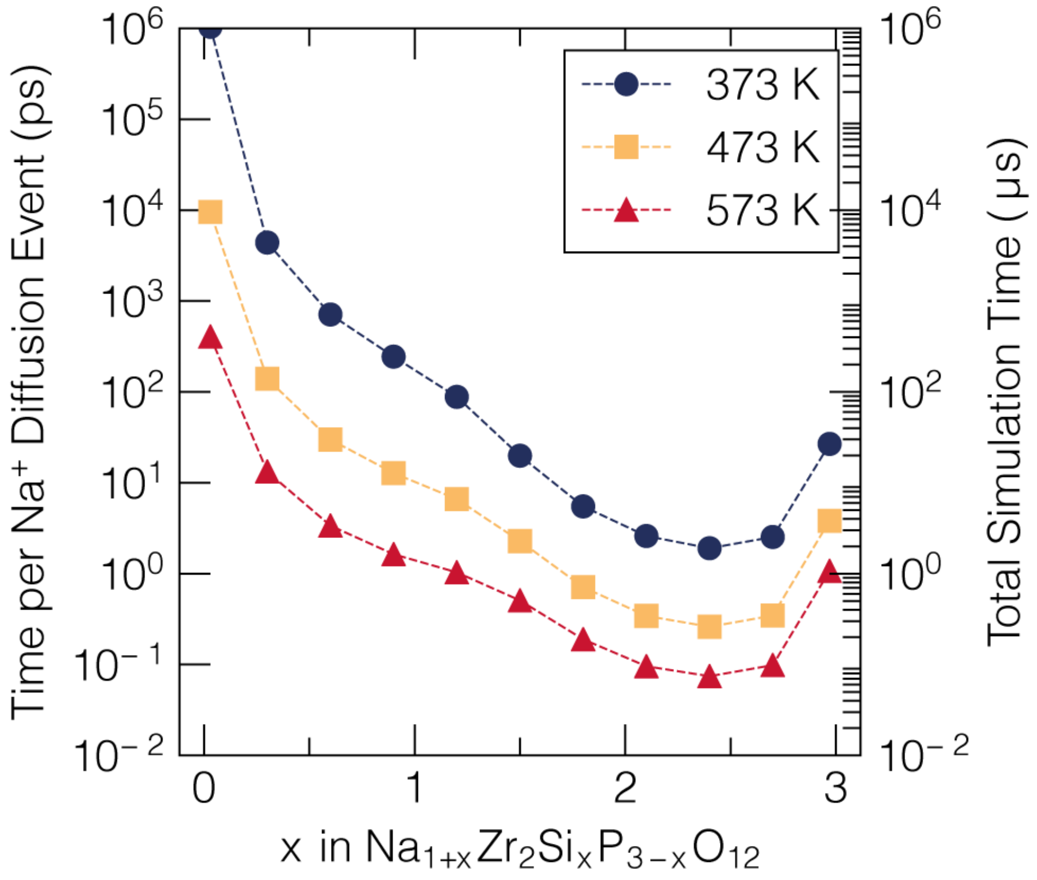

Supplementary Figure 5. Average time for each Na^+ diffusion event (in ps) and total simulation time at temperatures of 373, 473 and 573 K, respectively. Total time is defined as accumulation in Supplementary Equation 6 for all the kMC events.

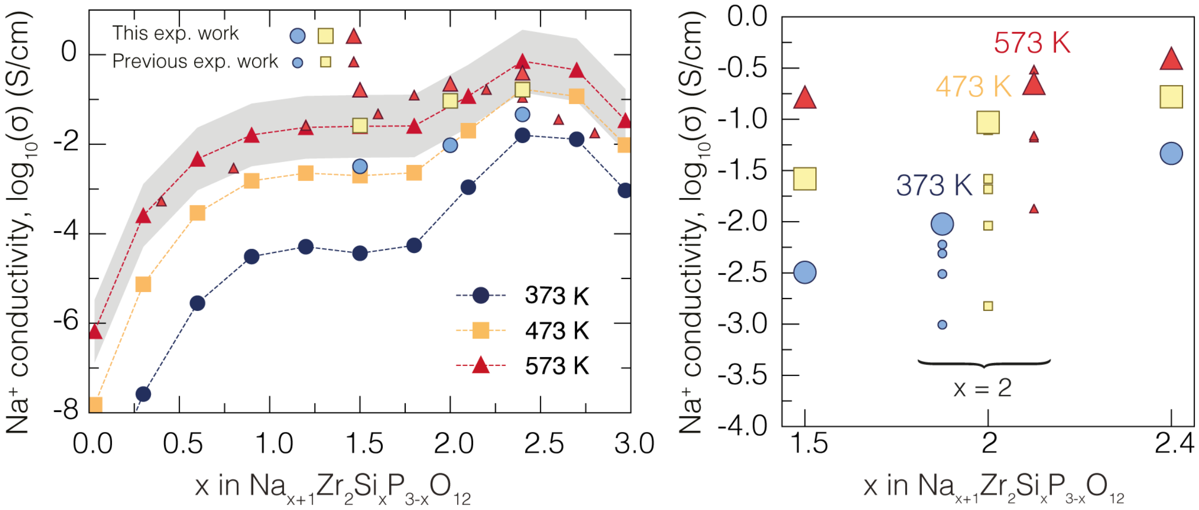

Supplementary Figure 6. Comparison of computed (large symbols without border) with our experimentally measured Na^+ conductivities (> 90% compacities, large symbols with border) or previous experimental data (small symbols with border) of $Na_{1+x}Zr_2Si_xP_{3-x}O_{12}$ (panel a) at variable temperatures, i.e., 373, 473 and 573 K.[1–3] Panel b shows a zoom in of our experimentally measured Na^+ conductivities (solid circles, squares and triangles) at 373, 473 and 573 K compared with previously measured conductivities at selected Na content (x = 1.5, 2 and 2.4).[1–3]

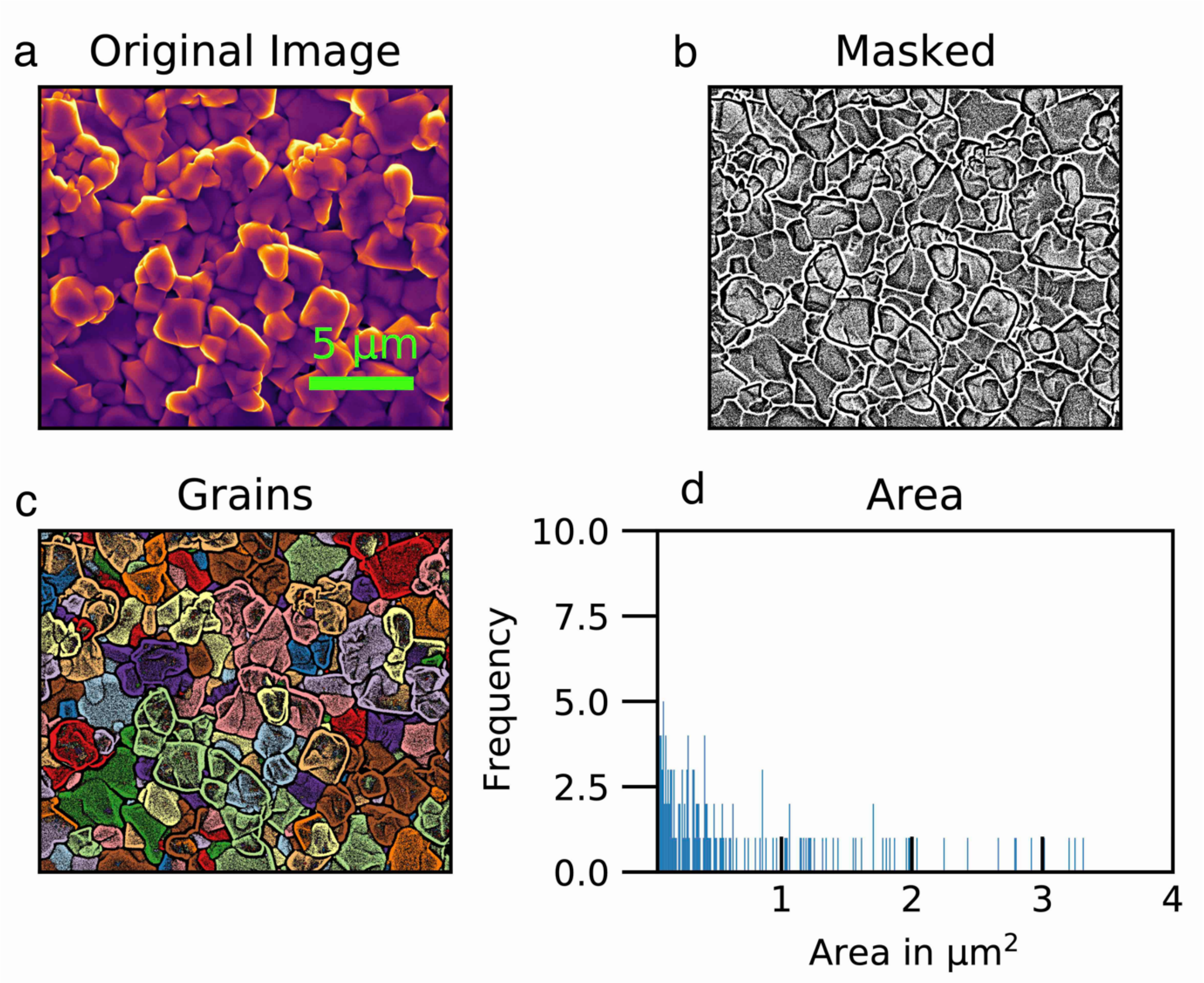

Supplementary Figure 7. SEM analysis of sintered pellet with composition $Na_{2.5}Zr_2Si_{1.5}P_{1.5}O_{12}$. Panel a shows the as recorded SEM picture. Panels b shows a filtered micrograph using an adaptive thresholding algorithm using a neighborhood area of 15x15 px^2. Panel c shows the NASICON grains isolated using the boundary detection algorithm. Panel d shows the resulting histogram of particle size. The average grain area for $Na_{2.5}Zr_2Si_{1.5}P_{1.5}O_{12}$ was found to be ~ 0.028 μm^2 with the maximum grain area of 9 μm^2 and minimum grain area of 4 x 10^{-4} μm^2.

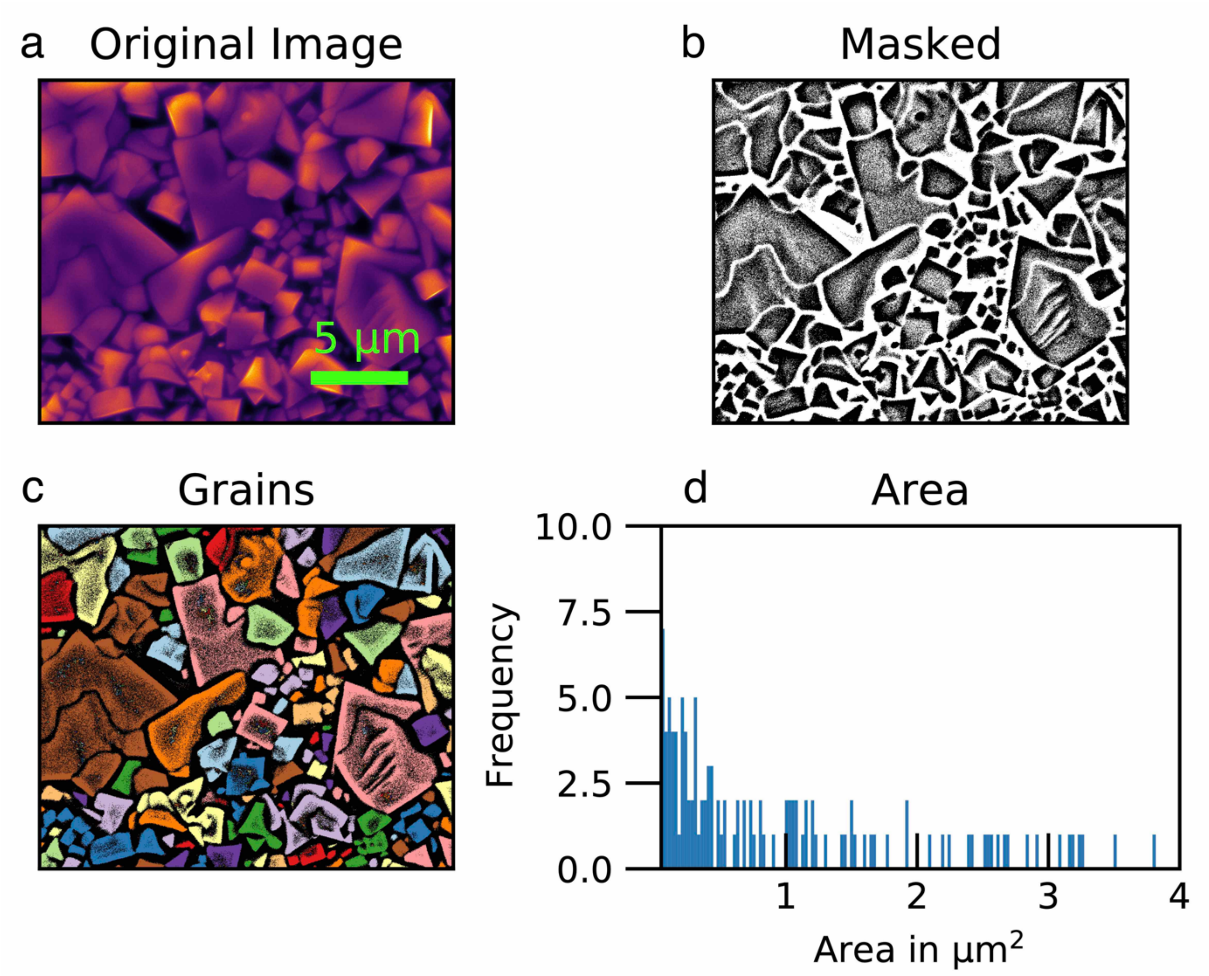

Supplementary Figure 8. SEM analysis of sintered pellet with composition $Na_3Zr_2Si_2P_1O_{12}$. Panel a shows the as recorded SEM picture. Panels b shows a filtered micrograph using an adaptive thresholding algorithm using a neighborhood area of 15x15 px^2. Panel c shows the NASICON grains isolated using the boundary detection algorithm. Panel d shows the resulting histogram of particle size. The average grain area for $Na_3Zr_2Si_2P_1O_{12}$ was found to be ~ 0.043 μm^2 with the maximum grain area of 24.8 μm^2 and minimum grain area of 4.6 x 10^{-4} μm^2.

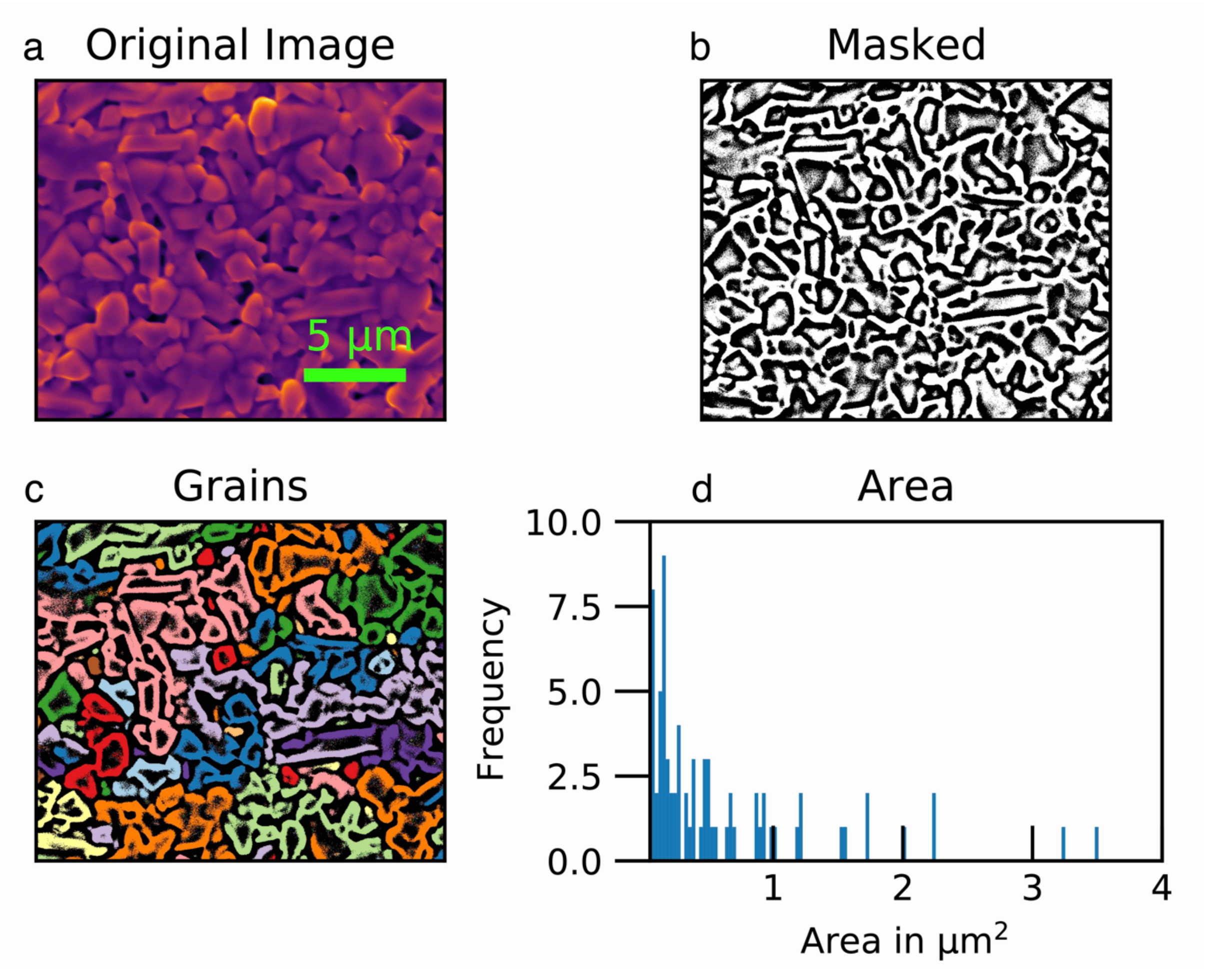

Supplementary Figure 9. SEM analysis of sintered pellet with composition $Na_{3.4}Zr_2Si_{2.4}P_{0.6}O_{12}$. Panel a shows the as recorded SEM picture. Panels b shows a filtered micrograph using an adaptive thresholding algorithm using a neighborhood area of 15x15 px^2. Panel c shows the NASICON grains isolated using the boundary detection algorithm. Panel d shows the resulting histogram of particle size. The average grain area for $Na_{3.4}Zr_2Si_{2.4}P_{0.6}O_{12}$ was found to be ~ 0.035 μm^2 with the maximum grain area of 28.5 μm^2 and minimum grain area of 4 x 10^{-4} μm^2.

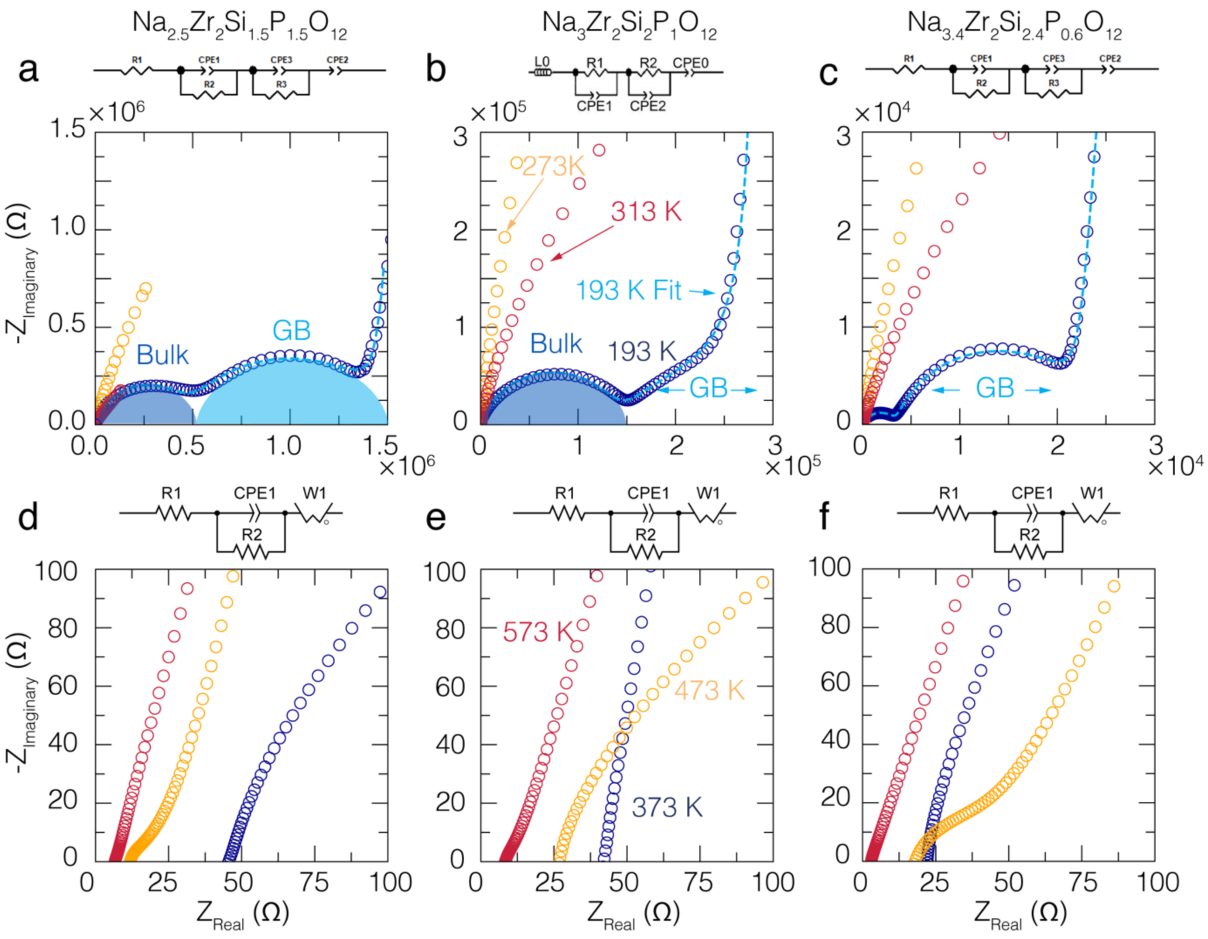

Supplementary Figure 10. Variable temperature impedance spectra of $Na_{1+x}Zr_2Si_xP_{3-x}O_{12}$ with nominal compositions (a, d) x = 1.5, (b, e) x = 2.0 (c, f) x = 2.4, respectively. The impedance spectra are obtained for different temperatures of ~193 K, ~273 K and 313 K and high-to-mid frequency (3 GHz – 1 Hz) for panel a-c, and ~373 K, ~473 K and 573 K for mid-to-low frequency (10 MHz – 0.1 Hz) panels d-f. The equivalent circuits are shown above each plot. Examples of fits of the impedance spectra are shown in panels a, b and c. The bulk and apparent grain boundary resistances are qualitatively indicated by the semicircles and arrows as a guide for the eye. In high-frequency low-to-moderate temperature AC Impedance measurements, two semicircles were observed, corresponding to bulk (high frequencies) and grain boundary (low frequencies) resistance contributions (see samples x =1.5 and x = 2.4 in Supplementary Figure 10a and Supplementary Figure 10c). Therefore, two in-parallel resistances (R) + constant phase elements (CPE) were used in series to model such phenomena (refer to insets in panels a-c). A CPE element describes the non-ideal capacitor behavior in solid state conductors. In Supplementary Figure 10a and Supplementary Figure 10c, R_1 represents the contact resistance. In Supplementary Figure 10b, the contact resistance element was substituted by an inductance element (L0 in inset). In Supplementary Figure 10a-c, a CPE element (CPE2 in panel a; CPE0 in panel b; and CPE2 in panel c) was adopted for the diffusion tail. For the x = 2.0 sample, the tail phenomenon at lower temperature (Supplementary Figure 10b) represents the grain boundary resistance that is hardly identifiable in the Nyquist plot, whereas the semicircle at high frequency corresponds to the bulk resistance. At higher temperatures: 373, 473 and 573 K, (Supplementary Figure 10d-e), both the bulk and grain boundary contributions become indistinguishable in all samples, thus only one R+CPE element was used in the equivalent circuit. In Supplementary Figure10d-f (x = 1.5, x = 2.0, x = 2.4), the equivalent circuit used was based on semicircle phenomenon, as seen in other reports,[3–7] with R1 the contact resistance, a CPE (CPE1) and R (R2) element in parallel representing the semi-circle, and a CPE for the diffusion tail. Since the CPE (modelling the diffusion tail) displays an n-value close to 0.5, a Warburg (W1) open element is used to better fit the diffusion tail. CPE, R, and inductor parameters obtained (and their relative errors) from fitting selected experimental spectra (at 197 K) in panel a, b and c are shown in Supplementary Table 10.

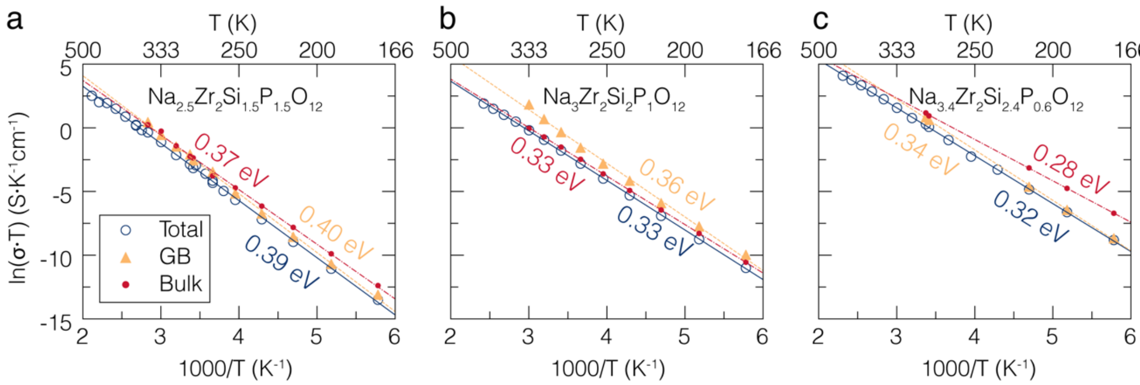

Supplementary Figure 11. Arrhenius plot vs. 1/Temperature as a function of temperature for $Na_{1+x}Zr_2Si_xP_{3-x}O_{12}$ with (a) x = 1.5, (b) x = 2.0 (c) x = 2.4, as extracted from high-frequency AC impedance spectroscopy. Bulk and apparent grain boundary (GB) contributions are shown separately.

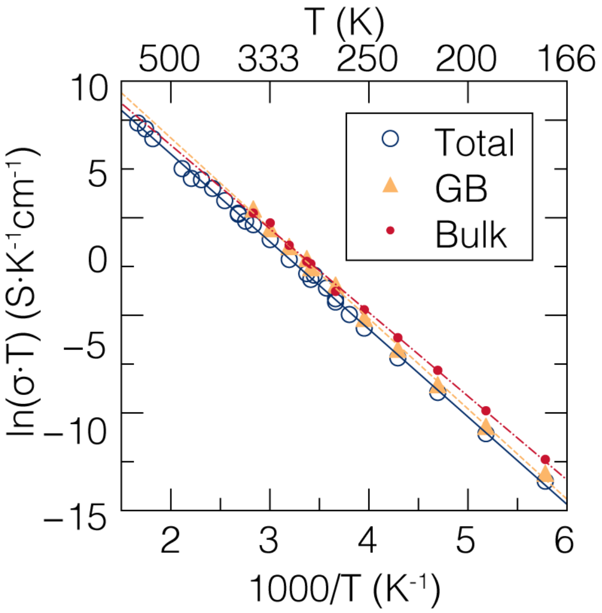

Supplementary Figure 12. High-temperature extrapolation of bulk and grain-boundary contributions to the total ionic conductivity in $Na_{2.5}Zr_2Si_{1.5}P_{1.5}O_{12}$.

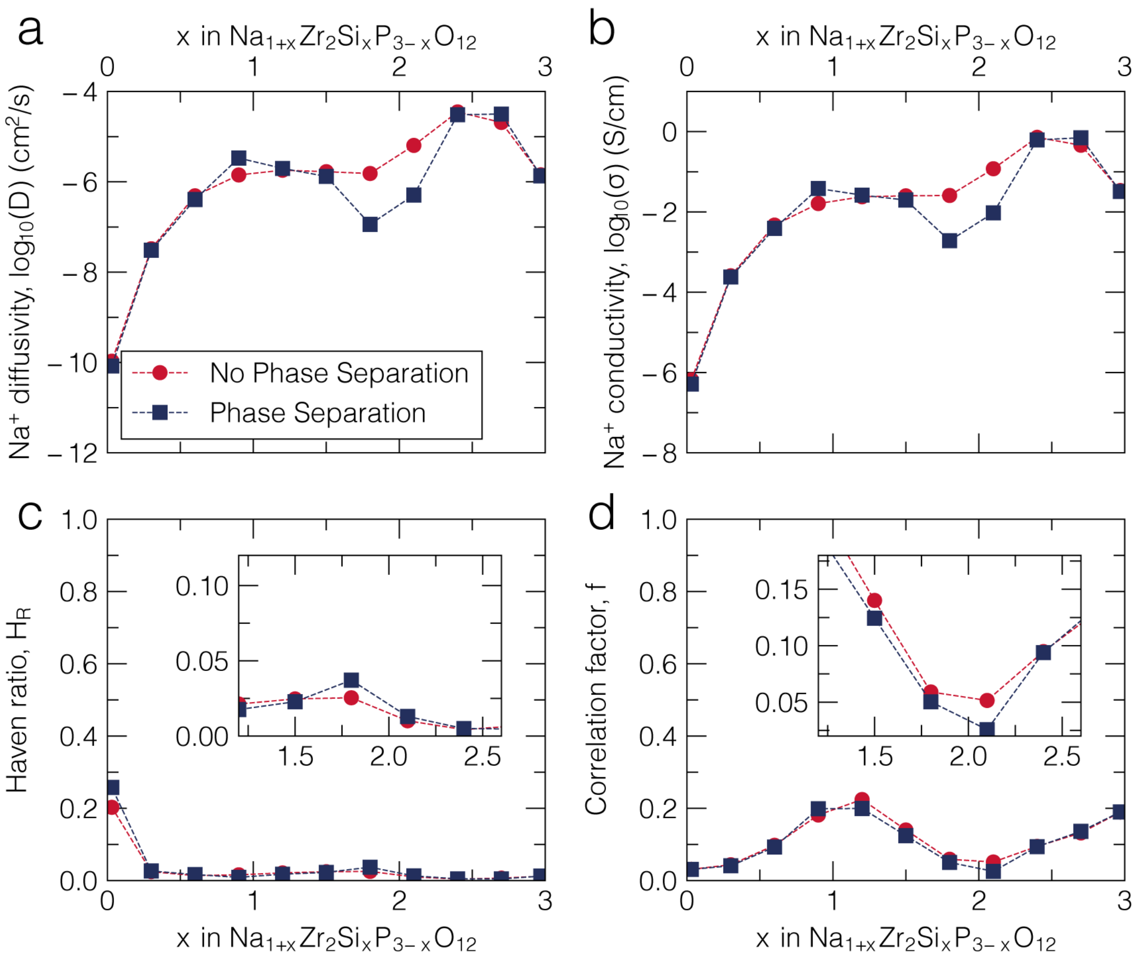

Supplementary Figure 13. Calculated Na^+ diffusivity (panel a), conductivity (panel b), Haven's ratio (panel **c**) and averaged correlation factor (panel d) from first principles kinetic Monte Carlo of $Na_{1+x}Zr_2Si_xP_{3-x}O_{12}$ at 573 K in regimes of phase separation (dark blue squares) or solid solution (red circles).

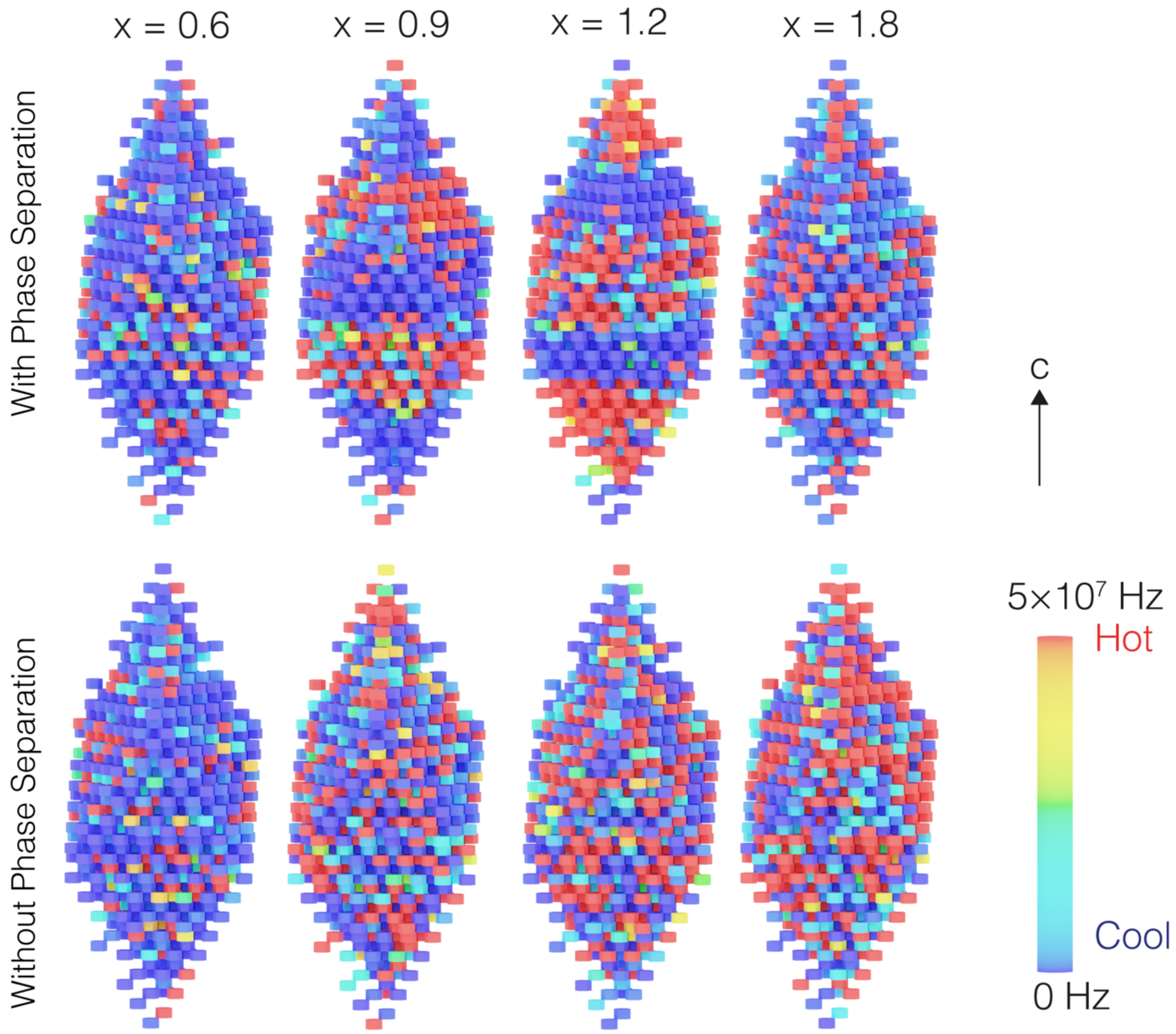

Supplementary Figure 14. Comparison of Na^{+} migration frequency in regimes of phase separation (top) and solid solution (bottom).

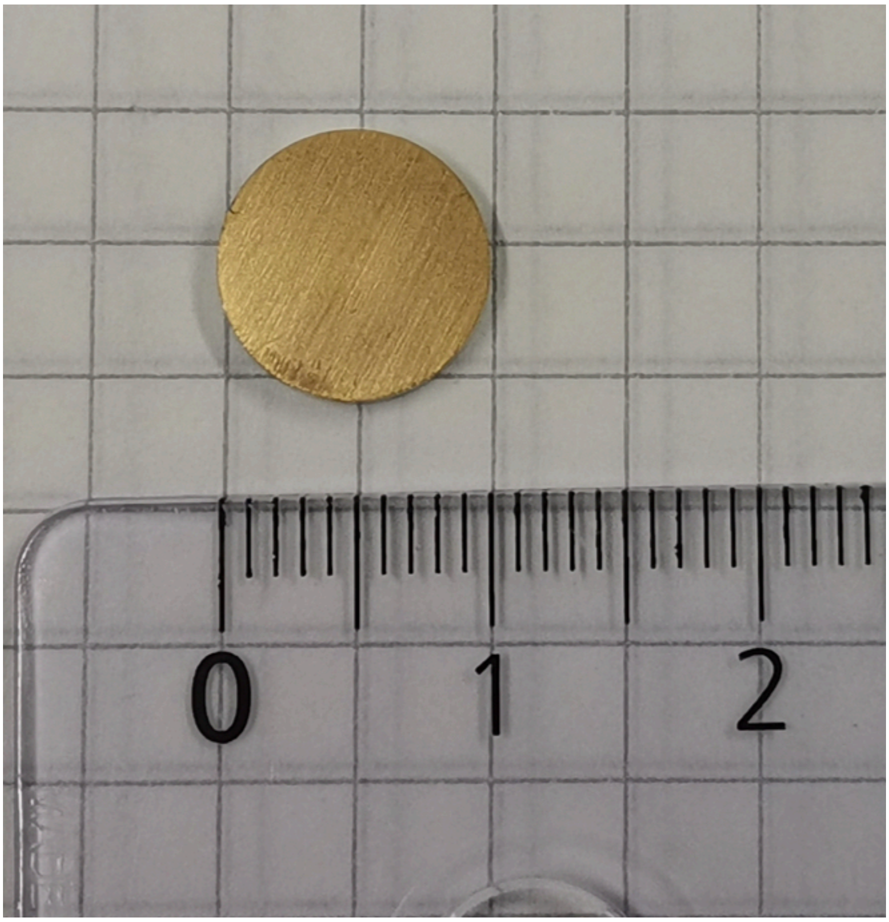

Supplementary Figure 15. Representative gold coated NASICON sample as used in the electrochemical impedance measurements.

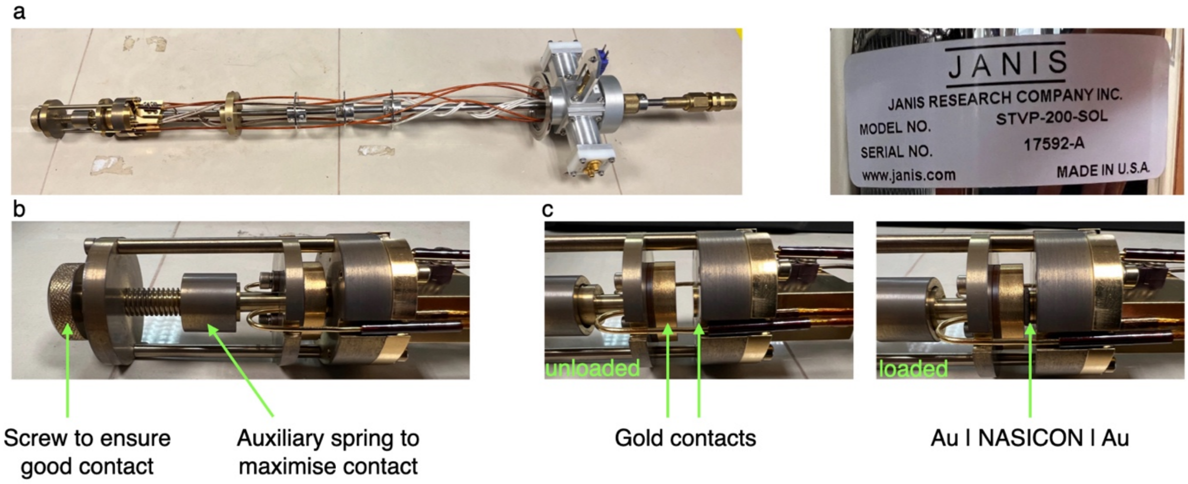

Supplementary Figure 16. Impedance cell, heating elements and a thermocouple as part of the Janis STVP-200-Sol cryostat setup used for the high-temperature impedance measurements. Panel a shows a longitudinal view of the 2-electrode impedance cell setup and the model of the cryostat. Panel b displays the screw and spring mechanism to load the sample in the cell. Panel c displays the gold contacts of the cell unloaded or loaded with a NASICON sample + carbon paper on each side.

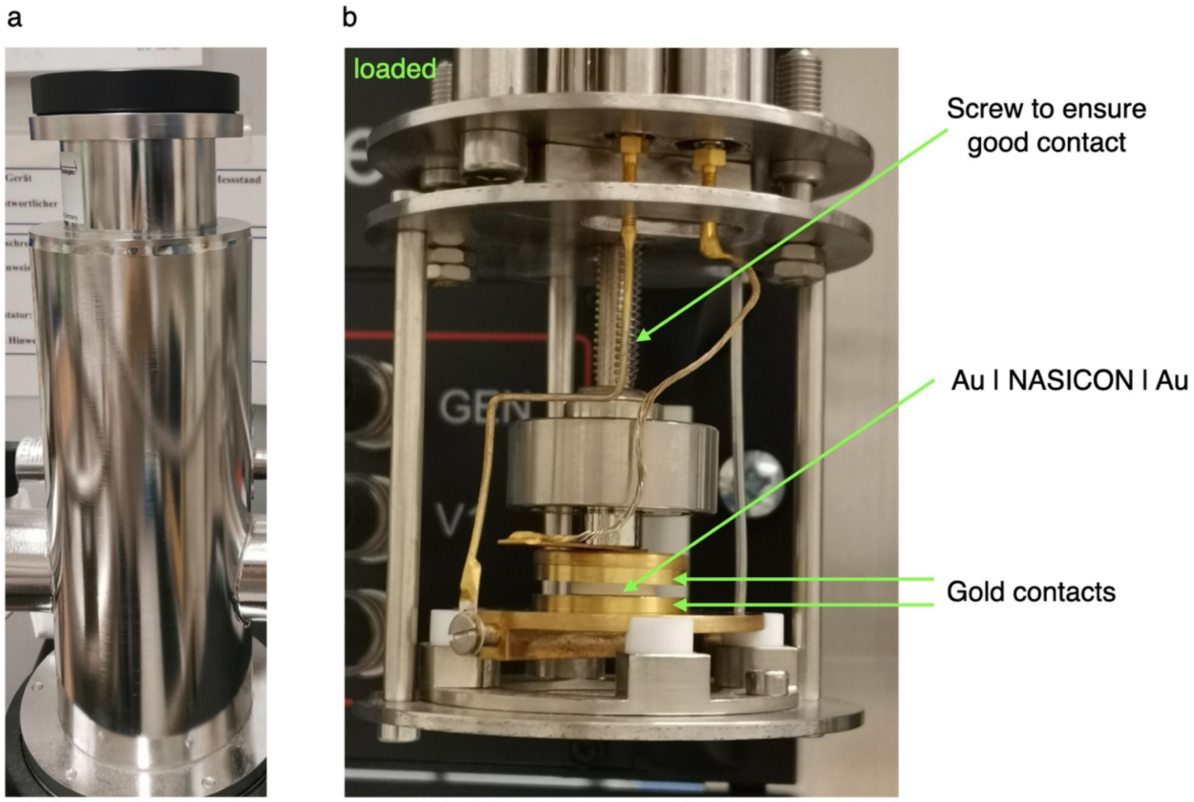

Supplementary Figure 17. Impedance cell, part of the cryostat setup used for the mid-to-low temperature (refer to main text) high-frequency impedance measurements. Panel a shows a longitudinal view of the impedance cell. Panel b displays the gold contacts of the 2-electrode impedance cell setup loaded with a NASICON sample in symmetric configuration Au | NASICON | Au.

Supplementary Tables

x	Configuration	Hop sequence	E_{KRA}	ΔE_{end}	$E_{barrier}$
0	0Si3P_0Si3P	First	236.2	446.5	458.3
		Second	236.1	446.9	458.1
2	0Si3P_0Si3P	First	495.9	651.3	821.5
		Second	395.7	791.7	791.5
2	0Si3P_1Si2P	Single Hop	484.4	413.4	691.1
2	1Si2P_0Si3P	Single Hop	390.8	536.1	658.8
2	0Si3P_2Si1P	Single Hop	396.8	73	433.2
2	2Si1P_0Si3P	Single Hop	398.0	48.8	422.4
2	0Si3P_3Si0P	First	321.7	-407.5	525.4
		Second	333.9	-666.5	667.2
2	3Si0P_0Si3P	First	125.8	-74.6	88.5
		Second	545.9	-269	411.5
2	1Si2P_1Si2P	First	302.9	43.5	324.7
		Second	311.1	43.7	332.9
2	3Si0P_1Si2P	First	182.5	-356.9	360.9
		Second	419.9	-625.7	732.7
2	2Si1P_2Si1P	First	341.7	-546.7	615
		Second	351.7	-546.8	625.1
2	2Si1P_3Si0P	Single Hop	394.0	114	451
2	3Si0P_3Si0P	First	311.9	-623.9	623.9
		Second	312.6	-625.2	625.2
3	3Si0P_3Si0P	Single	153.2	306.3	306.3
**Charge Balanced Structures**					
2	0Si3P_2Si1P	Single Hop	445	-34.4	462.2
2	0Si3P_3Si0P	First	299.5	412.6	505.8
		Second	373.3	-123	496.3
2	3Si0P_0Si3P	First	406.2	-68.8	440.6
2	1Si2P_1Si2P	First	101.6	169.3	186.2
		Second	271.2	169.1	186.6
2	1Si2P_2Si1P	First	353.4	-50.11	378.5
		Second	400.2	192.99	496.7
2	2Si1P_1Si2P	First	180.4	362.6	361.7
		Second	1044.4	-304.7	892.1

Supplementary Table 1. Calculated NEB results: E_{KRA}, ΔE_{end} and $E_{barrier}$. ΔE_{end} = E[Na@Na(2)] – E[Na@Na(1)]. Units are in meV

Site Index	Element	x	y	z
0	Na/Va	-2.9381	0.214774	-1.848417
1	Na/Va	-1.65505	-2.437083	1.848417
2	Na/Va	-1.283051	2.651856	1.848417
3	Na/Va	1.283051	-2.651856	-1.848417
4	Na/Va	1.65505	2.437083	-1.848417
5	Na/Va	2.9381	-0.214774	1.848417
6	Si/P	-3.23615	0.301463	1.848417
7	Si/P	-1.87915	-2.651856	-1.848417
8	Si/P	-1.357	2.953319	-1.848417
9	Si/P	1.357	-2.953319	1.848417
10	Si/P	1.87915	2.651856	1.848417
11	Si/P	3.23615	-0.301463	-1.848417

Supplementary Table 2. Element and the Cartesian coordinates (Å) of each site in the local structure.

Index	Type	Site Index	max_length (Å)	min_length (Å)	Multiplicity
0	point	(0,)	0.0	0.0	6
1	point	(6,)	0.0	0.0	6
2	pair	(0, 1)	4.727	4.727	6
3	pair	(0, 3)	5.103	5.103	6
4	pair	(0, 5)	6.956	6.956	3
5	pair	(0, 6)	3.71	3.71	6
6	pair	(0, 7)	3.056	3.056	6
7	pair	(0, 8)	3.162	3.162	6
8	pair	(0, 9)	6.492	6.492	6
9	pair	(0, 10)	6.543	6.543	6
10	pair	(0, 11)	6.196	6.196	6
11	pair	(6, 7)	4.922	4.922	6
12	pair	(6, 9)	5.629	5.629	6
13	pair	(6, 11)	7.478	7.478	3
14	triplet	(0, 1, 2)	5.103	4.727	6
15	triplet	(0, 1, 6)	4.727	3.162	6
16	triplet	(0, 1, 7)	4.727	3.056	6
17	triplet	(0, 3, 4)	5.103	5.103	2
18	triplet	(0, 3, 7)	5.103	3.056	6
19	triplet	(0, 6, 7)	4.922	3.056	6
20	triplet	(0, 6, 8)	4.922	3.162	6
21	triplet	(0, 7, 8)	5.629	3.056	6
22	triplet	(6, 7, 8)	5.629	4.922	6
23	triplet	(6, 9, 10)	5.629	5.629	2

Supplementary Table 3. Point, pair, and triplet orbits generated from the cut-off values of 10 Å and 6 Å for the pair and triplet terms respectively for both the E_{KRA} and ΔE_{end} in local structure. The local structure and site index are defined in Supplementary Table 4.

Cluster #	Cluster Index	Type	KECI
1	0	point	10.5
2	1	point	4.08
3	4	pair	–11.26
4	6	pair	–4.06
5	8	pair	–7.21
6	12	pair	2.36
7	13	pair	–2.71
8	14	triplet	12.42
9	22	triplet	7.41
10	Empty Cluster	–	339.11

Supplementary Table 4. Non-zero clusters and their KECI (in units of meV/multiplicity) obtained from local cluster expansion of the E_{KRA}.

Cluster #	Cluster Index	Type	KECI
1	0	point	66.38
2	1	point	7.45
3	4	pair	–10.01
4	6	pair	–28.74
5	8	pair	–39.12
6	10	pair	–29.00
7	12	pair	115.68
8	20	triplet	–3.6
9	21	triplet	–5.76
10	23	triplet	–89.18
11	Empty Cluster	–	137.19

Supplementary Table 5. Non-zero clusters and their KECI (in units of meV/multiplicity) obtained from local cluster expansion of the ΔE_{end}.

x	Mass (g)	Thickness (cm)	Surface area (cm^2)	Compacity (%)	ZrO_2 (wt. %)
1.5	0.3033	0.125	1.043	90.7	2.4
2.0	0.2105	0.108	0.554	90.4	10.5
2.4	0.3628	0.133	0.864	95.8	0.11

Supplementary Table 6. NASICON pellet characteristics, including the mass (accuracy ±0.0001g) and thickness of the samples (accuracy ±0.01mm), the measured surface area. Thickness and surface areas are used to compute the volume of the pellets, which is then used to estimate the relative density of the sintered pellets to the NASICON bulk. The compacity of the pellets indicates the deviation of the pellet density from the NASICON theoretical density (at specific Na contents), which is assumed as 100 %. Supplementary Table 6 also reports the amount of ZrO_2 impurities in wt. %. derived from the Rietveld refinements of the powder X-ray diffraction presented in the main text.

Species	Wyckoff	x	y	z	U_{iso}	Occ.
Zr	12c	0.00000	0.00000	0.14901(10)	0.012	1.000
P	18e	0.2977(7)	0.00000	0.25000	0.020	0.500
Si	18e	0.2977(7)	0.00000	0.25000	0.020	0.500
O	36f	0.1759(10)	–0.0337(11)	0.1974(4)	0.009	1.000
O	36f	0.1911(7)	0.1691(10)	0.0847(5)	0.020	1.000
Na(1)	6b	0.00000	0.00000	0.00000	0.047	0.670
Na(2)	18e	0.628(3)	0.00000	0.25000	0.169	0.576

Supplementary Table 7. Structural parameters obtained from the Rietveld refinements of the PXRD diffractogram of $Na_{2.5}Zr_2Si_{1.5}P_{1.5}O_{12}$. Space group $R\overline{3}c$, a = 8.9918(6) and c = 22.9983(19) Å.

Species	Wyckoff	x	y	z	U_{iso}	Occ.
Zr	8f	0.0996(3)	0.2367(4)	0.0509(3)	0.0072	1.000
P1	4e	0.0000	0.067(2)	0.25000	0.0023	0.333
P2	8f	0.3576(8)	0.1209(12)	0.2870(15)	0.0023	0.333
Si1	4e	0.0000	0.067(2)	0.25000	0.0023	0.667
Si2	8f	0.3576(8)	0.1209(12)	0.2870(15)	0.0023	0.667
O1	8f	0.1560(15)	0.437(3)	0.258(3)	0.0063	1.000
O2	8f	0.4345(16)	0.455(3)	0.098(3)	0.0063	1.000
O3	8f	0.2747(15)	0.241(2)	0.240(2)	0.0063	1.000
O4	8f	0.3707(16)	0.133(3)	0.129(3)	0.0063	1.000
O5	8f	0.4415(18)	0.156(2)	0.420(3)	0.0063	1.000
O6	8f	0.0663(18)	0.160(3)	0.214(3)	0.0063	1.000
Na(1)	4d	0.25000	0.25000	0.50000	0.1706	1.000
Na(2)	4e	0.50000	0.899(3)	0.25000	0.0090	1.000
Na(3)	8f	0.7828(18)	0.074(4)	0.689(3)	0.0090	0.500

Supplementary Table 8. Structural parameters obtained from the Rietveld refinements of the PXRD diffractogram of $Na_3Zr_2Si_2P_1O_{12}$. Space group $C2/c$, a = 15.6844(15), b = 9.0493(9), c = 9.2320(7) Å, and β = 124.157(4) ∘.

Species	Wyckoff	x	y	z	U_{iso}	Occ.
Zr	8f	0.09838(18)	0.2471(6)	0.05620(16)	0.0038	1.000
P1	4e	0.00000	0.0590(16)	0.25000	0.0007	0.200
P2	8f	0.3669(6)	0.1085(12)	0.2743(12)	0.0007	0.200
Si1	4e	0.00000	0.0590(16)	0.25000	0.0007	0.800
Si2	8f	0.3669(6)	0.1085(12)	0.2743(12)	0.0007	0.800
O1	8f	0.1395(11)	0.4067(16)	0.2296(18)	0.0050	1.000
O2	8f	0.4330(10)	0.4605(19)	0.0610(19)	0.0050	1.000
O3	8f	0.2746(10)	0.1921(19)	0.2279(18)	0.0050	1.000
O4	8f	0.3772(9)	0.1338(16)	0.1397(19)	0.0050	1.000
O5	8f	0.4484(13)	0.1813(17)	0.4335(20)	0.0050	1.000
O6	8f	0.0867(12)	0.1506(19)	0.2556(18)	0.0050	1.000
Na(1)	4d	0.25000	0.25000	0.50000	0.1938	1.000
Na(2)	4e	0.50000	0.890(3)	0.25000	0.0007	0.76
Na(3)	8f	0.8034(10)	0.066(2)	0.6948(15)	0.0007	0.785

Supplementary Table 9. Structural parameters obtained from the Rietveld refinements of the PXRD diffractogram of $Na_{3.4}Zr_2Si_{2.4}P_{0.6}O_{12}$. Space group $C2/c$, *a* = 15.7344(4), *b* = 9.1001(2), and *c* = 9.1990(2) Å, and β = 124.3397(13) ∘.

$Na_{1+x}Zr_2Si_xP_{3-x}O_{12}$	x = 1.5	x = 2.4	x = 2.0	
**R1** (Ω)	0.00 (0.00)	0.00 (0)	**L0** (H)	1E-12
**CPE1** (F)	2.263E-10 (4.93)	1.941E-9 (5.48)	**CPE1** (F)	1.6E-11 (10.0)
**R2** (Ω)	491110 (1.43)	3461 (0.44)	**R1** (Ω)	1.50E5 (3.4)
**CPE3** (F)	5.827E-9 (4.53)	2.6328E-8 (2.91)	**CPE2** (F)	9.1E-9 (6.04)
**R3** (Ω)	2.0168E6 (1.30)	19016 (0.56)	**R2** (Ω)	9.0E4 (0.35)
**CPE2** (F)	1.1117E-7 (3.43)	2.008E-7 (2.49)	**CPE0** (F)	6.9E-7 (10.1)

Supplementary Table 10. Equivalent circuit parameters, i.e. resistor (R), constant phase element (CPE) and inductance (L) obtained from fitting selected low-temperature (197 K) impedance spectra (see blue lines) of $Na_{1+x}Zr_2Si_xP_{3-x}O_{12}$ sample (with nominal compositions x = 1.5, x = 2.0 and x = 2.4), as shown in Supplementary Figure 10a-c. Labels of R, CPE and L elements is given in the insets of Supplementary Figure 10a-c. Errors in % are given in brackets. R1 in x =1.5 and x =2.4 arises from the cable and contact interactions and were constrained to 0 Ω in the fit, whereas for composition x = 2, L0 was set to $1x10^{-12}$ H.

Supplementary Note 1

Quantum mechanical density functional theory (DFT) calculations were performed using the Vienna ab initio Simulation Package (VASP).[8,9] Projected augmented wave (PAW) potentials[10,11] were used with the following electrons treated explicitly: Na $3s^1$, Zr $4s^2 4p^6 4d^2 5s^2$, Si $3s^2 3p^2$, P $3s^2 3p^3$ and O $2s^2 2p^4$. The exchange-correlation energy was calculated using the strongly constrained and appropriately normed (SCAN) functional.[12] A 2×2×1 Monkhorst-Pack[13] *k*-point mesh and a 520 eV kinetic energy cutoff were used to integrate the DFT total energy in the 1st Brillouin zone and expand the multielectron wave-functions of the $Na_{1+x}Zr_2Si_xP_{3-x}O_{12}$ (NASICON) structures. These settings were employed for the structure optimization of the NASICON in the hexagonal conventional unit cell (6 f.u.), whereas for the pseudo-cubic structure (8 f.u.) at low Na content (mentioned below) the total energy was only sampled at the Γ-point.

Three Na concentrations were computed: x = 0, 2 and 3, where Na diffuses along Na(1)→Na(2)→Na(1), Na(2)→Na(1)→Na(2), and Na(1)→Na(2), respectively. A charge-neutral Na vacancy was created for each structure to enable these migrations. Selected energy formation to create these vacancies were added to the energy barrier fitting (see Method section in the main text). For x = 0, a large pseudo-cubic supercell structure (8 f.u.) was used and obtained by transforming the primitive trigonal cell (2 f.u.) using a matrix: [[1, 1, -1], [-1, 1, 1], [1, -1, 1]]. For x = 2 and 3, the most stable configurations[14] were taken (6 f.u.). At x = 2, different local Si/P configurations were also calculated. Structures with charge-neutral vacancies at initial and final images in the diffusion path were fully optimized until the interatomic forces were less than 0.01 eV/Å. These images are interpolated and used for nudged elastic band simulation as described in the next section.

Supplementary Note 2

Climbing image nudged elastic band (CI-NEB) calculations were performed using the method by Henkelman et al.[15] Calculated NEB results are shown in Supplementary Figure 1, Supplementary Figure 2, Supplementary Figure 3, and Supplementary Table 1. The NEB calculations were carried out using the SCAN functional and following the computational settings indicated in the main manuscript.[12]

The different local environments were created by replacing the Si/P from the two triangles shown in Supplementary Figure 1a. Our NEB plots follow the convention of αSiβP_γSiδP. In this nomenclature, the values of α, β, γ and δ are derived by counting the occupation of Si and P in the two Si and P triangles (Supplementary Figure 1a). The atomic species (Si or P) for sites numbered 1-2-3 are set α and β indices, and sites 4-5-6 are set γ and δ indices, as shown in Supplementary Figure 1b. For example, 0Si3P_3Si0P correspond to a migration unit, where sites 1-2-3 are occupied by Si only and sites 4-5-6 by P only.

As per Supplementary Figure 1a, the calculated barrier follows two distinct migration events indicated in Supplementary Table 1 as first Na(2)→Na(1) and second Na(1)→Na(2), which are captured by the NEB barriers. Thus, we compute the complete migration barrier Na(2)→Na(1)→Na(2) by averaging these two events (i.e., Na(2)→Na(1) and Na(1)→Na(2)). The only exception is $Na_4Zr_2(SiO_4)_3$ where all Na(2) sites are occupied, in this case we have only explored half path Na(1)→Na(2). Supplementary Figure 1a shows the two migration paths and an example of nomenclature of migration environment.

Supplementary Note 3

As mentioned in Figure 1 in the main text, a local cluster expansion (LCE) model is constructed by searching the nearest-neighbour Na and Si/P sites around a Na(1) site with a cut-off of 5 Å. The local structural motif used in the model is listed in Supplementary Table 1. Within the local structure, cut-offs of 10 Å and 6 Å were used for searching the pairs and triplets, respectively, which results in the generation of 24 distinct orbits. These orbits are used to generate the Hamiltonian to determine the E_{KRA} and the endpoint energy as shown in Supplementary Equation 1:

$$E = V_0 + \sum_{orbit} V_{orbit}\,\phi_{orbit};$$ **Supplementary Equation 1**

where V_0 and V_{orbit} are called empty cluster interaction and kinetic effective cluster interactions (KECI), which are the coefficients to be determined. The information of the points, pairs and triplets terms are shown in Supplementary Supplementary **Table 3**. E is either kinetically resolved activation barrier[16] (E_{KRA}) or energy difference between Na located at Na(1) and Na(2) sites (ΔE_{end} = E[Na@Na(2)] – E[Na@Na(1)]). ΔE_{end} and E_{KRA} are fitted separately using 2 sets of V_0 and V_{orbit} (Supplementary Table and Supplementary Table) and the diffusion barrier between Na(1) and Na(2) sites can be calculated as below:

$$E_{barrier} = \frac{\sigma_{Na(2)} - \sigma_{Na(1)}}{4} \Delta E_{end} + E_{KRA};$$ **Supplementary Equation 2**

σ is the site occupation (σ[Na/Si] = -1 and σ[Va/P] = 1). $\sigma_{Na(1)}$ and $\sigma_{Na(2)}$ are the Na site occupations at the Na(1) and Na(2) sites for each diffusion event. An orbit is a collection of all symmetrically equivalent clusters (points, pairs, and triplets), and ϕ_{orbit} is called the correlation vector of an orbit. This can be evaluated by summing up the products of the occupation of each site within each cluster using Supplementary Equation 3:

$$\phi_{orbit} = \sum_{cluster \in orbit} \prod_{site \in cluster} \sigma_{site}.$$ **Supplementary Equation 3**

Then a lasso fitting is performed with an L1 regularization parameter of α = 1.8.[17] Supplementary Table shows the 10 KECIs identified (1 empty term, 2 point terms, 5 pairs, and 2 triplets). Supplementary Table shows the 11 ECIs (1 empty term, 2 point terms, 5 pairs and 3 triplets) required to fit the DFT values of ΔE_{end}. A comparison between the fitted results and the computed from DFT is shown in Supplementary Figure 4.

Supplementary Note 4

Rejection-free kinetic Monte Carlo (kMC) simulations were performed using the method described in van der Ven et al.,[16,18] using the LCE Hamiltonian (V_0 and V_{orbit} in Supplementary Equation 1). Only Na migration is considered in this study and Si/P/O are kept fixed. The migration probability of each Na diffusion event can be determined by:

$$\Gamma[event] = \nu^{*} exp\left[\frac{-E_{barrier}[event]}{k_B T}\right]; \qquad \textbf{Supplementary Equation 4}$$

where the $E_{barrier}$ for this event can be calculated using Supplementary Equation 2, and ν^* is related to the vibrational entropy of the image at the energy maximum and minimum along the migration path, which is taken as 5×10^{12} Hz in this study, and k_B is Boltzmann constant. Then an event is chosen by generating a random number ρ between 0 and 1 (Supplementary Equation 5):

$$\frac{1}{\Gamma_{tot}}\sum_{m=1}^{k-1}\Gamma_m < \rho \le \frac{1}{\Gamma_{tot}}\sum_{m=1}^{k}\Gamma_m; \qquad \textbf{Supplementary Equation 5}$$

where Γ_{tot} is the summation of the probability for all events and m is the index of the event to be chosen. The time Δt for this Na migration event can be computed using Supplementary Equation 6:

$$\Delta t = \frac{-\ln(\xi)}{\Gamma_{tot}}. \qquad \textbf{Supplementary Equation 6}$$

where ξ is a random number between 0 and 1.

Each kMC simulation started from a configuration (8×8×8 supercell, 1024 f.u.) generated from canonical Monte Carlo at 1500 K using the model from Deng et al,[14] and was equilibrated for ~ 2 million (2,048,000) steps and followed by ~ 12 million (12,288,000) sampling steps. 11 compositions (x = 0.03, 0.3, 0.6, 0.9, 1.2, 1.5, 1.8, 2.1, 2.4, 2.7, 2.97) were computed, and 50 configurations were simulated for each composition to obtain better statistics. kMC simulations were performed at 373, 473 and 573 K.

All Na^+ were tracked during kMC simulations and Na^+ diffusivity D, Na^+ tracer diffusivity D^*, Haven's ratio H_R, and averaged correlation factor f, can be evaluated from Na^+ trajectories. All computed quantities (D, D^*, H_R and f), as well as Na^+ occupancy are arithmetically averaged for 50 structures at each composition for each temperature. D is computed using Supplementary Equation 7:

$$D = \frac{1}{6tN}\left(\sum_{i=1}^{N}\vec{r}_i(t)\right)^2; \qquad \textbf{Supplementary Equation 7}$$

where is the displacement vector of a Na^+ with an index i at after time t and N is the total number of Na^+ in the simulation cell. The correlation between different Na^+ can be measured by Haven's ratio calculated as Supplementary Equation 8.

$$H_R = \frac{D^*}{D}; \qquad \textbf{Supplementary Equation 8}$$

where D^* can be evaluated as Supplementary Equation 9.

$$D^{*} = \frac{1}{6tN}\left(\sum_{i=1}^{N}[\vec{r}_i\,(t)]^2\right);$$ **Supplementary Equation 9**

Averaged correlation factor f measures the correlation between successive Na^+ jumps for a single Na^+, then averaged by the total number of Na^+ in a structure, as in Supplementary Equation 10.

$$f = \frac{1}{N}\sum_{i=1}^{N}\frac{1}{M_i a^2}\sum_{j=1}^{M_i}[\vec{r}_j(t)]^2.$$ **Supplementary Equation 10**

where indices *i* and *j* refer to different Na^+ and different jumps for a single Na^+, respectively. M_i is the total number of hopping for each Na^+ and is the averaged hopping distance (3.4778 Å). f is taken as 0 when there is no diffusion after simulation ends.

Supplementary Note 5

The pellet dimensions are shown in Supplementary Table 6. Supplementary Table 7, Supplementary Table 8 and Supplementary Table 9 list the structural parameters obtained from the Rietveld refinements of the PXRD diffractograms of $Na_{2.5}Zr_2Si_{1.5}P_{1.5}O_{12}$, $Na_3Zr_2Si_2PO_{12}$ and $Na_{3.4}Zr_2Si_{2.4}P_{0.6}O_{12}$ presented in the main text.

Supplementary Figure 10 shows the AC impedance spectra measured at high-frequency (3 GHz – 1 Hz) and low temperature (panels a-c), as well as mid-to-low frequency (10 MHz – 0.1 Hz) and high temperature (panels d-f). Details on the setup of these measurements are given in the methodology section of the main text. Appropriate equivalent circuits are also shown. Dashed blue lines in Supplementary Figure 10 indicate an example of such fit. The equivalent circuits used to fit the low-to-moderate temperature, high-frequency AC impedance are shown in Supplementary Figure 10a-c, whereas the high-temperature measurements are shown in Supplementary Figure 10d-e.

The equivalent circuits used to extract the total conductivity (resistivity), bulk and apparent grain boundary conductivities are shown in of each $Na_{1+x}Zr_2Si_xP_{3-x}O_{12}$ sample are shown in Supplementary Figure 10. Supplementary Table 10 shows the resistance, capacitance and inductance values of resistors, constant phase elements and inductors of each equivalent circuit as obtained from fitting selected impedance data of Supplementary Figure 10a-c.The tail phenomenon observed in sample x = 2.0 is common in solid electrolytes, e.g. $Li_7La_3Zr_2O_{12}$.[19] At low temperatures, in sample x = 2.0, the apparent grain boundary resistance is smaller than the bulk resistance (Supplementary Figure 10b), which signifies that grain boundaries do not impede Na-ion transport.

Supplementary Figure 11 shows Arrhenius plots derived from the high-frequency (3 GHz to 0.1 Hz) AC impedance data in the temperature range of 433 to 173 K of $Na_{1+x}Zr_2Si_xP_{3-x}O_{12}$ compositions: x = 1.5 (panel a), x = 2.0 (b) and x = 2.4 of the sintered pellets in Supplementary Table 5. These measurements can resolve the bulk, E_a(bulk) and grain boundary E_a(GB) activation energies from the total activation energy E_a(total), which quantify the resistive effects of grain boundaries on the overall Na-ion transport in $Na_{1+x}Zr_2Si_xP_{3-x}O_{12}$. As temperature increases in Supplementary Figure 11a and Supplementary Figure 11b, the grain boundaries conductivities approach monotonically the values of bulk conductivities, and their differences appears negligible for any measurement above 333 K (~60 °C). Therefore, it is safe to say that at temperature above 333 K (~60 °C) the total ionic conductivity is only dominated by Na-ion transport in the bulk of the NaSICON particles. In $Na_3Zr_2Si_2P_1O_{12}$ grain boundaries appear to help Na^+ transport, so the limiting factor appears the bulk resistance.

The highest regime of ion transport in NaSICONs with compositions for $1.8 < x < 2.5$ is achieved at temperature above 150 °C, and beyond monoclinic-to-rhombohedral phase transition. Indeed, separating bulk from grain boundary phenomena at high temperatures is not a trivial task, hitting the capabilities of high-frequency impedance spectrometers. Because for NaSICON compositions with $1.8 < x < 2.5$ show a phase transition from monoclinic-to-rhombohedral,[1,20] a change in slopes in the Arrhenius curves at high temperature is observed at high temperatures (see Figure 4b of the main text). In contrast, at composition $x < 1.8$ and $x > 2.5$, $Na_{1+x}Zr_2Si_xP_{3-x}O_{12}$ remains always in the rhombohedral phase.[14,20] To this end, we can only extrapolate grain boundaries effects at temperatures higher than ~300 K (Supplementary Figure 12) using the impedance data of $Na_{2.5}Zr_2Si_{1.5}P_{1.5}O_{12}$ (x = 1.5), which is guaranteed to remain rhombohedral in the full temperature range (–100 to 300 °C). Supplementary Figure 12 demonstrates that the grain boundary contributions to the total conductivity is insignificant.

The available body of facts presented in Supplementary Figures 10-12 suggest that grain boundary contributions to the total conductivity will be only important at low temperatures, but not at temperatures above 60 °C (100 °C, 200 °C and 300 °C) central to this manuscript. An exception to this is trend is $Na_3Zr_2Si_2P_1O_{12}$, where grain boundary resistance helps Na^+ transport.

Supplementary References

1. Goodenough, J. B., Hong, H. Y.-P. & Kafalas, J. A. Fast Na^+-ion transport in skeleton structures. *Materials Research Bulletin* **11**, 203–220 (1976).
2. Deng, Y. *et al.* Crystal Structures, Local Atomic Environments, and Ion Diffusion Mechanisms of Scandium-Substituted Sodium Superionic Conductor (NASICON) Solid Electrolytes. *Chem. Mater.* **30**, 2618–2630 (2018).
3. Ma, Q. *et al.* Scandium-Substituted $Na_3Zr_2(SiO_4)_2(PO_4)$ Prepared by a Solution-Assisted Solid-State Reaction Method as Sodium-Ion Conductors. *Chem. Mater.* **28**, 4821–4828 (2016).
4. Grady, Z. M., Tsuji, K., Ndayishimiye, A., Hwan-Seo, J. & Randall, C. A. Densification of a Solid-State NASICON Sodium-Ion Electrolyte Below 400 °C by Cold Sintering With a Fused Hydroxide Solvent. *ACS Appl. Energy Mater.* **3**, 4356–4366 (2020).
5. Bohnke, O. Conductivity measurements on nasicon and nasicon-modified materials. *Solid State Ionics* **122**, 127–136 (1999).
6. Liu, S. *et al.* Ce-Substituted Nanograin $Na_3Zr_2Si_2PO_{12}$ Prepared by LF-FSP as Sodium-Ion Conductors. *ACS Appl. Mater. Interfaces* **12**, 3502–3509 (2020).
7. Naqash, S., Ma, Q., Tietz, F. & Guillon, O. $Na_3Zr_2(SiO_4)_2(PO_4)$ prepared by a solution-assisted solid state reaction. *Solid State Ionics* **302**, 83–91 (2017).
8. Kresse, G. & Furthmüller, J. Efficient iterative schemes for ab initio total-energy calculations using a plane-wave basis set. *Physical Review B* **54**, 11169–11186 (1996).
9. Kresse, G. & Hafner, J. Ab initio molecular dynamics for liquid metals. *Physical Review B* **47**, 558–561 (1993).
10. Blöchl, P. E. Projector augmented-wave method. *Physical Review B* **50**, 17953–17979 (1994).
11. Kresse, G. & Joubert, D. From ultrasoft pseudopotentials to the projector augmented-wave method. *Physical Review B* **59**, 1758–1775 (1999).
12. Sun, J., Ruzsinszky, A. & Perdew, J. P. Strongly Constrained and Appropriately Normed Semilocal Density Functional. *Physical Review Letters* **115**, 036402 (2015).
13. Monkhorst, H. J. & Pack, J. D. Special points for Brillouin-zone integrations. *Physical Review B* **13**, 5188–5192 (1976).
14. Deng, Z. *et al.* Phase Behavior in Rhombohedral NaSiCON Electrolytes and Electrodes. *Chemistry of Materials* **32**, 7908–7920 (2020).
15. Henkelman, G., Uberuaga, B. P. & Jónsson, H. A climbing image nudged elastic band method for finding saddle points and minimum energy paths. *The Journal of Chemical Physics* **113**, 9901–9904 (2000).
16. Van der Ven, A., Deng, Z., Banerjee, S. & Ong, S. P. Rechargeable Alkali-Ion Battery Materials: Theory and Computation. *Chemical Reviews* **120**, 6977–7019 (2020).
17. Nelson, L. J., Hart, G. L. W., Zhou, F. & Ozoliņš, V. Compressive sensing as a paradigm for building physics models. *Phys. Rev. B* **87**, 035125 (2013).
18. Van der Ven, A., Ceder, G., Asta, M. & Tepesch, P. D. First-principles theory of ionic diffusion with nondilute carriers. *Physical Review B* **64**, 184307 (2001).
19. Cojocaru-Mirédin, O. *et al.* Quantifying lithium enrichment at grain boundaries in $Li_7La_3Zr_2O_{12}$ solid electrolyte by correlative microscopy. *Journal of Power Sources* **539**, 231417 (2022).
20. Hong, H. Y.-P. Crystal structures and crystal chemistry in the system $Na_{1+x}Zr_2Si_xP_{3-x}O_{12}$. *Materials Research Bulletin* **11**, 173–182 (1976).
